# “Lessons Learned” Preventing Recurrent Ischemic Strokes through Secondary Prevention Programs: A Systematic Review

**DOI:** 10.3390/jcm10184209

**Published:** 2021-09-17

**Authors:** Clare McGarvey Lambert, Oluwaseyi Olulana, Lisa Bailey-Davis, Vida Abedi, Ramin Zand

**Affiliations:** 1 Geisinger NeuroScience Institute, Geisinger Health System, Danville, PA 17822, USA; clare.lambert@yale.edu (C.M.L.); OOlulana@som.geisinger.edu (O.O.); 2 Yale New Haven Hospital, Department of Neurology, New Haven, CT 06510, USA; 3 Department of Population Health Sciences, Geisinger Health System, Danville, PA 17822, USA; ldbaileydavis@geisinger.edu; 4 Department of Molecular and Functional Genomics, Geisinger Health System, Danville, PA 17822, USA; vabedi@geisinger.edu; 5 Department of Public Health Sciences, College of Medicine, The Pennsylvania State University, Hershey, PA 16802, USA

**Keywords:** recurrent stroke, secondary prevention, cerebrovascular disease

## Abstract

Recurrent ischemic strokes are a cause of significant healthcare burdens globally. Patients with uncontrolled vascular risk factors are more likely to develop recurrent ischemic strokes. This study aims to compile information gained from current secondary prevention programs. A pre-defined literature search strategy was applied to PubMed, SCOPUS, CINAHL, and Google Scholar databases, and studies from 1997 to 2020 were evaluated for quality, study aims, and outcomes. The search produced 1175 articles (1092 after duplicates were removed) and titles were screened; 55 titles were retained for the full-text analysis. Of the remaining studies, 31 were retained for assessment, five demonstrated long-term effectiveness, eight demonstrated short-term effectiveness, and 18 demonstrated no effectiveness. The successful studies utilized a variety of different techniques in the categories of physical fitness, education, and adherence to care plans to reduce the risk of recurrent strokes. The lessons we learned from the current prevention programs included (1) offer tailored care for underserved groups, (2) control blood pressure, (3) provide opportunities for medication dosage titration, (4) establish the care plan prior to discharge, (5) invest in supervised exercise programs, (6) remove barriers to accessing care in low resource settings, and (7) improve the transition of care.

## 1. Introduction

Stroke is a global public health concern in both developed and developing nations, spanning multiple healthcare delivery systems [1]. The annual incidence of stroke in the United States is approximately 800,000 and one-quarter of these are recurrent strokes [2,3]. While clinicians and scientists have been encouraged by a 36.2% global decrease in deaths due to stroke and an 11.3% decrease in age-standardized stroke incidence rates from 1990 to 2016 [4,5], there remains room for improvement in terms of preventing recurrent strokes, which continue to be a source of ongoing disability [1,6]. Finding a way to accurately establish the risk of having a recurrent ischemic stroke remains difficult [7] and makes it challenging to plan and evaluate the effect of secondary prevention efforts [8,9].

There are a number of modifiable risk factors such as hypertension, diabetes, dyslipidemia, smoking, and physical inactivity that increase the risk of recurrent strokes and therefore often serve as the focus of recurrent stroke prevention programs [10]. The purpose of this systematic review was to identify peer-reviewed recurrent ischemic stroke prevention programs and analyze them in an effort to better inform the development and implementation of future programs. This review characterized the methods employed by effective studies, compared to unsuccessful studies, to generate a set of recommendations that may help elucidate what elements future successful stroke prevention programs should incorporate.

## 2. Methods

*Search Strategy*: This review was written in alignment with the “Enhancing the QUAlity and Transparency of Health Research (EQUATOR)” [11] and the “Preferred Reporting Items for Systemic Reviews and Meta-Analyses (PRISMA; Table S1)” [12] guidelines. The search engines utilized included PubMed, SCOPUS, CINAHL, and Google Scholar. The search strategy was created by team members, in conjunction with the librarian at Geisinger Health Center, and was defined as: “stroke recurrence prevention” OR “stroke prevention initiative” OR “stroke prevention program” OR “stroke prevention trial” OR “stroke education program” OR “stroke education initiative” OR “stroke education trial” AND (“secondary prevention” OR “recurrent stroke” OR “stroke recurrence”). Limitations included “research papers”, “full text available”, and “English language”.

*Eligibility Criteria*: Two reviewers independently evaluated the titles and abstracts of the retrieved articles and screened the full texts based on the predefined inclusion/exclusion criteria. In case of any disagreement, the final decision was made through consultation with a third reviewer. Studies of interventional programs, feasibility protocol, and single-arm studies (no control group) were included. Only studies that targeted patients with prior stroke/transient ischemic attack (TIA), their caregivers, or healthcare providers were obtained for this review. Studies that focused on the general public (i.e., people without a stroke or TIA), studies without a clear intervention, studies without a clear primary outcome measure, and studies that focused solely on the efficacy of pharmacotherapies were excluded.

*Data Collection and Data Definitions*: Intervention title or acronym, authorship, year of publication, sample size, study design, study location, target population, level of intervention, delivery method, intervention leader, study strategy, and effectiveness were extracted and harmonized (Table 1). The intervention level was defined as the target population for which the study was designed to serve, based on the categorization system described by Cleary et al. 2012, in their discussion of multi-level interventions [13]. Level 1 interventions targeted individuals (i.e., patients with stroke or TIA) and Level 2 interventions targeted physicians and healthcare providers. Level 2 interventions could involve supplementary education for providers, simplifying workflows, removing barriers to providing care, or could target interpersonal relationships between providers and clients. Within this framework, Level 3 interventions targeted healthcare organizations and hospitals and Level 4 targeted communities [13]. In this review no Level 3 or 4 interventions, or multi-level interventions, were obtained through the search strategy.

The study objectives and results sections of each study were assessed. The study objective was categorized into increasing physical activity, improving education, and aiding in adherence to the care plan. Studies that utilized only education or only physical activity were categorized as such, all other strategies were grouped into adherence to the care plan, this included studies that focused on medication adherence, motivational interviewing, support groups, nurse driven objective metric reporting (such as blood pressure or cholesterol levels), adherence counselling, pillboxes, patient calendars, text message reminder services, and the identification and management of critical deviations from care targets.

The authors’ assessment of effectiveness was used to guide our categorization into “effective” versus “non-effective” categories. Some studies were deemed effective, even if statistical significance was not reached, when the authors concluded the intervention was effective and had a plausible explanation for the lack of statistical significance in their results. Studies were also assessed on their longevity of effectiveness. If positive results were established between zero- and six-months post-randomization, they were deemed to have short-term effectiveness, whereas if they maintained positive results for greater than six months post-randomization, they were deemed to have long-term effectiveness. For example, some studies performed their assessment immediately after applying their intervention, and therefore had a minimum time of zero months for short-term effectiveness; however, most other short-term effective studies utilized between one and three months as the minimum time to assess effectiveness [14,15].

## 3. Results

The predefined search strategy was run in March of 2020 and produced 1175 articles and 11 articles were identified through cross-reference checking. After the removal of duplicates, 1092 study titles were screened. Most titles (*n* = 1027) were eliminated due to lack of relevance and 55 were retained for the full-text analysis (see Supplemental Figure S1). Of the remaining studies, 31 were retained for discussion (Table 1 and Table 2, Supplemental Table S2). Several studies in their pilot stages focused only on outlining methodology and assessing feasibility; therefore, effectiveness could not be determined at this time. These studies are summarized in the Supplemental Materials (Table S2). The search strategy did not generate any Level 3 or 4 studies.

The most common study design was the randomized control trial (RCT, *n* = 27), followed by the cluster randomized control design (*n* = 3), pre-and post-test (*n* = 2), non-randomized control trial (*n* = 2), single-arm feasibility study (*n* = 2), randomized feasibility study (*n* = 1), and mixed methods (*n* = 1). Studies were most frequently conducted in Europe (*n* = 11), followed by the United States (*n* = 8), Asia (*n* = 4), Australia and New Zealand (*n* = 3), Africa (*n* = 2), the Middle East (*n* = 1), and Latin America (*n* = 1). One study was conducted inter-continentally between China, Poland, Estonia, and Denmark. Frequently utilized outcome measures were blood pressure (BP), medication adherence, knowledge scores, metabolic equivalent (MET) minutes, the 6-min walk test (6MWT), quality of life (QoL) scales, low-density lipoprotein cholesterol (LDL-C), recurrent stroke or readmission for a vascular event, depression scales (i.e., Hospital Anxiety and Depression Score, HADS) and mobility scales (i.e., modified Rankin score, mRS) (see Supplemental Table S3). Feasibility and pilot studies used mainly the rate of recruitment and feasibility as primary outcomes (Table 2).

Of the 31 completed experimental studies, 13 were effective and 18 were deemed not effective (Table 3). Of the 13 effective studies, five demonstrated long-term effectiveness (significant improvement in outcome measure at >6 months post-randomization) and eight demonstrated short-term effectiveness (at ≤6 months post-randomization). The three main intervention strategy categories were improving adherence to the care plan (utilized by 16/31 studies), improving education (14/31), and increasing physical activity (7/31). Several studies (11/31) used more than one core intervention strategy and therefore received more than one classification: two studies mixed education and physical activity, eight mixed education and improving adherence to the care plan, two mixed education and physical activity, one mixed physical activity and improving adherence to the care plan, and no program combined all three core strategies. The studies utilized an individual-level (Level 1) intervention approach 97% of the time (Table 4). Of the few Level 2 interventions, two focused solely on adherence to the care plan and one combined adherence to care plan with education.

**Figure 1:**
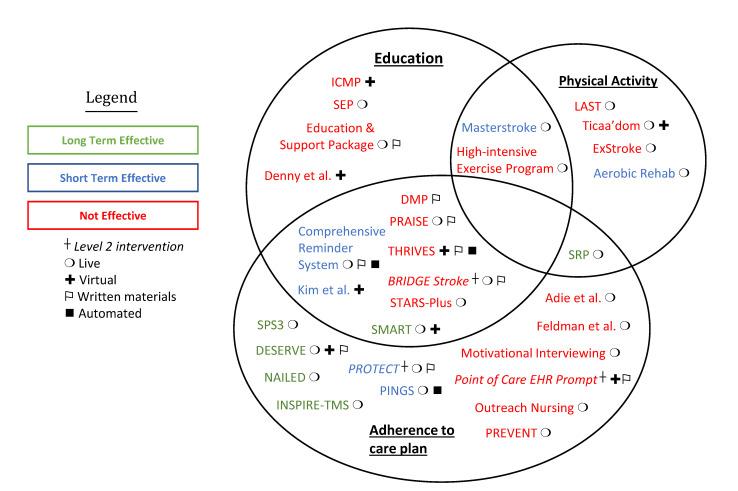
Provides a visual aid for navigating which studies utilized adherence to the care plan, physical activity, and education as their primary outcome and if the setting was live, virtual, written materials, automated, Level 1, or Level 2. Red studies were not effective, blue were short-term effective, and green were long-term effective, as labeled in the figure legend. Many studies combined two outcomes, but no studies utilized all three.

Studies that focused on adherence to care plan strategies were the only group of studies that demonstrated long-term effectiveness (SMART, DESERVE, NAILED, SPS3, INSPiRE-TMS, and SRP) [16,17,18,19,20,21]. SMART combined adherence to the care plan with education [16]. SRP combined adherence to the care plan with physical activity [21]. DESERVE, NAILED, SPS3, and INSPiRE-TMS focused solely on adherence to the care plan [17,18,19,20]. Masterstroke combined education with exercise and was able to demonstrate short-term effectiveness, making it the only study of this nature to be effective [15]. Equally as many programs focusing solely on adherence to the care plan or inclusive combinations were deemed not effective (Adie et al. and Feldman et al. Motivational Interviewing, Point of Care Electronic Health Record (EHR) prompt, Outreach Nursing, PREVENT, DMP, STARS-Plus, PRAISE, THRIVES, BRIDGE Stroke) [6,22,23,24,25,26,27,28,29,30,31]. Most programs that focused on education or education plus physical activity were not effective (SEP, ICMP, Education and Support Package, High-Intensive Exercise Program, Denny et al.) [32,33,34,35,36]. Additionally, programs that focused solely on physical activity were mostly not effective (Ticaa’dom, ExStroke, LAST) [37,38,39]. Figure 1 and Supplementary Table S1 include further detailed information about the included studies, strategies, and outcomes.

Live/in-person strategies were effective in a number of instances (11 out of 25; Comprehensive Reminder System, PROTECT, SRP, Masterstroke, NAILED, Aerobic Rehab, PINGS, DESERVE, SMART, INSPiRE-TMS, SPS3) [14,15,16,17,18,19,20,21,40,41,42]; however, many of the studies were not (14 out of 25; SEP, PRAISE, LAST, ExStroke, Ticaa’dom, High-Intensive Exercise Program, PREVENT, STARS Plus, Education and Support Package, BRIDGE Stroke, Outreach Nursing and Motivational Interviewing, Adie et al. and Feldman, et al.) [6,22,23,24,25,27,28,30,33,35,36,37,38,39]. Studies that utilized a virtual component of delivery were effective in three cases (SMART, DESERVE, Kim et al.) and not effective in five cases (ICMP, THRIVES, Point of Care EHR Prompt, Ticaa’dom, Denny et al.) [16,17,25,30,33,36,42,43]. Studies that complemented their strategy with written materials were more often unsuccessful (Education and Support Package, DMP, PRAISE, THRIVES, BRIDGE Stroke, Point of Care EHR Prompt) than successful (Comprehensive Reminder System, DESERVE, PROTECT) [14,17,23,26,29,30,31,35,43]. Only three studies had an automated component: Comprehensive Reminder System and PINGS were short-term effective, whereas THRIVES was not effective [31,40,41,43]. Only three studies utilized a Level 2 approach: two were not effective (Point of Care EHR and BRIDGE Stroke) and one was effective in the short-term (PROTECT) [14,23,26].

### 3.1. Long-Term Effective Studies (>6 Months)

NAILED, SPS3, INSPiRE-TMS, SRP, SMART, and DESERVE demonstrated long-term effectiveness [16,17,18,19,20,21]. NAILED focused on follow-up counseling telephone calls and medication titration opportunities if targets were not being met in terms of BP and LDL-C. In the control group, targets and abnormal blood/BP results were forwarded to the primary care provider (PCP), but were not necessarily followed up on, and phone calls were made to check-in, but no counseling took place. The study demonstrated a significant decrease in BP and LDL-C, regardless of education level, whereas the control group only saw improvements in the higher education groups [18]. The SPS3 trial looked at using a target of <130 mmHg in post-stroke patients (versus < 140 mmHg) and used 3-monthly outpatient visits with medication titration to ensure this goal was being met. As a result, they observed a downward trend in percent recurrent stroke per patient-year at the one-year follow-up and the intervention group additionally saw a reduction in intracerebral hemorrhagic strokes [19]. The INSPiRE-TMS trial also focused on managing critical deviations from target values through referral to the emergency department (ED), complemented by increased outpatient follow-ups that focused on motivational interviewing and lifestyle risk factor management plan development. They reported improvements in physical activity levels and the stair-climbing test. Additionally, the intervention group demonstrated improved smoking abstinence, antiplatelet use, Hemoglobin A1C (HbA1C), and body mass index (BMI) at one year; however, the changes did not persist to three years [20]. SRP utilized a modified cardiac rehabilitation program for post-stroke patients [21]. The intervention was conducted in a group setting rather than individually and there was no inclusion of the perceived dyspnea scale, otherwise, the rehabilitation program remained largely the same as traditional post-cardiac intervention rehabilitation [21,44]. This study demonstrated improved mobility, daily activity, and MET-minutes at 120-days post-randomization. Up to one year later they identified a significant reduction in unadjusted mortality which suggested the control group had a nine times higher chance of dying than the intervention group [21]. SMART focused on improving adherence to antiplatelet, antihypertensive, anti-diabetic, anticoagulation, and statin medications, with the secondary aim of reducing recurrent strokes and all-cause mortality, through a mixed live/virtual intervention [16]. Their program provided an in-person interactive education session prior to discharge that focused on lifestyle modifications, as well as access to an interactive educational website. They observed improved adherence to statins through this initiative at both 6 and 12 months. However, there was no improvement in antiplatelet, antihypertensive, anti-diabetic, or anticoagulant use [16]. DESERVE was a live plus virtual-based program with a focus on adherence to the care plan; however, it also included a workbook (written materials) as part of the program [17]. They reported up to a 10 mmHg drop in systolic BP in a subset of patients who identified as Hispanic; however, the overall group systolic BP did not differ from controls [17].

### 3.2. Short-Term Effective Studies

The Comprehensive Reminder System, PROTECT, Masterstroke, Aerobic Rehab, PING, SMART, and Kim et al. measured short-term effectiveness and were deemed effective [14,15,16,40,41,42,45]. The Comprehensive Reminder System provided face-to-face education before discharge along with a comprehensive calendar handbook, followed by a health belief telephone education session and automated text message reminders regarding clinic visits, lifestyle modifications, medication adherence, and cautioning against engaging in poor health behaviors [41,43]. The program reported a nearly 10-point reduction in systolic BP, as well as an improved Health-Promoting Lifestyle Profile (HPLP) score, increased physical activity, improved nutrition, and improved medication adherence at three months; however, there was no difference in smoking and alcohol use [43] The PROTECT study was one of the few Level 2 interventions [14]. Physicians on the stroke ward were provided with education on the importance of antithrombotic therapy, statins, anti-hypertensives, and smoking cessation for post-stroke care. They were also provided with premade order forms to streamline the process of ensuring appropriate prescriptions and education were established in patients’ care plans prior to discharge [14]. After the implementation, there was increased statin, angiotensin-converting enzyme inhibitors (ACEi), angiotensin receptor blockers (ARBs), and thiazide diuretic prescriptions as well as improved rates of patient education prior to discharge compared to rates reported prior to the study onset. There was no effect on antithrombotic therapy initiation and no long-term follow-up to see if the improved pre-discharge care plan was adhered to later along in the recovery process [14].

Kim et al. provided education videos, through a series of nine web-based stroke education sessions which allowed patients and caregivers to repeatedly view video resources [45]. The web tool allowed for some degree of customization of education based on self-rated healthcare behaviors [45]. This program demonstrated a significant improvement in exercise, diet, and health motivation. However, there was no difference in LDL-C, smoking, drinking, or medication adherence [45]. PINGS looked at providing post-stroke care in Ghana; they provided patients with pillboxes and BP monitors and followed up by sending automated reinforcement text messages every day to remind patients to take their medications and to check their BP at home every three days [40]. The interval between reminder text messages decreased after patients demonstrated two weeks of 100% adherence. This was a pilot study, and not powered to detect a significant difference, but has demonstrated a trend towards an increasing proportion of participants meeting the BP target of <140/90 and improved medication possession rates [40].

Masterstroke provided patients with one-hour teaching and one-hour exercise sessions weekly for nine weeks and the group demonstrated reduced salt intake, improved stroke knowledge, and improved fitness (measured via the Timed Up and Go Test, i.e., “TUG”) at three months post-randomization; however, there was no difference in 6MWT scores [15]. Aerobic Rehab engaged in a supervised exercise program and demonstrated improved 6MWT and Met-minutes in participants; however, no significant decrease in BP was identified [42].

### 3.3. Studies That Were Not Effective

Denny and colleagues created a five-minute educational stroke video played for patients at the bedside prior to discharge [32]. Patients took a knowledge quiz before the video, immediately after watching the video (to assess knowledge acquisition), and 30-days post-stroke (to assess knowledge retention). The average score only improved by one point on the 10-question non-validated knowledge quiz after watching the video; however, this improvement was sustained at 30 days. The study was categorized as not short-term effective due to the lack of clinical significance associated with one point of improvement [32]. In EP, High-intensive Exercise Program, and LAST, the standard of care (control group condition) was more effective than the intervention, for some outcome measures [33,36,39]. SEP and High-intensive Exercise Program demonstrated improved caregiver social functioning and improved patient mental health in the control group, while other metrics remained unchanged by the intervention [33,36]. The only difference detected in the LAST trial was an improved TUG test in the control group at 18 months post-randomization [39].

THRIVES and Feldman et al. reported both short and long-term decreased BP (8.3–11.6 mmHg on average); however, the control and intervention groups did not differ from one another [24,31]. The PRAISE trial intervention group trended towards a slightly larger magnitude of BP reduction; however, this was also not statistically different from the control group [30,38]. Ticaa’dom observed an increased walking distance in the 6MWT in the intervention and control group from baseline at both 6 and 12 months; however, the intervention group did not perform better than controls [37]. Adie et al. reported improved medication knowledge after the intervention, but no difference in systolic BP, total cholesterol, or medication adherence [22]. ExStroke was unable to demonstrate a significant change in the Physical Activity Scale for the Elderly (PASE) at 24 months. Nonetheless, a sub-analysis of those who attended all sessions suggested improved PASE values; however, once pre-intervention PASE was controlled for, the change was no longer present [38].

The Point of Care EHR Prompt, a Level 2 intervention, did not identify any improvement in BP, statin, anticoagulant, or antiplatelet prescriptions after installing the program, which was intended to highlight to physicians which high-risk patients needed preventive care, and 25% of doctors did not access the software throughout the study period [26]. STARS-Plus also had a generally low uptake of their program materials and did not demonstrate a significant change in the 30-day versus 365-day 12-item Short Form Survey (SF-12) score [6]. PREVENT did not demonstrate significant improvement in hypertension, except for a slightly lower diastolic BP in the intervention group; however, the limited clinical significance of this single finding resulted in the study being categorized as not effective in the long-term [28]. DMP did not have the power to demonstrate statistically significant findings; however, there was a trend towards favoring the program in patients over 65 who were non-smokers without chronic kidney disease [29]. ICMP did not demonstrate any significant change in stroke knowledge following their multimedia computer-based education session [34]. The Education and Support Package did not demonstrate statistically significant improvement in most of their target measures; however, the intervention group showed significant improvement in self-efficacy in accessing stroke information, feeling informed, and satisfaction with information received. Each of these was only assessed with single items on a scale, so the clinical relevance of these changes is questionable [35].

BRIDGE Stroke did not demonstrate any significant difference between interventions and controls on their primary composite adherence outcome, which was the sum of antithrombotic use, deep vein thrombosis prophylaxis use, tissue plasminogen activator (tPA) door to needle time, dysphagia screening performed, rehabilitation assessment performed, statin prescriptions written, anticoagulation prescriptions written, and smoking cessation education being provided [23]. They identified a slight predilection towards hemorrhagic transformation in the intervention group [23]. Outreach Nursing did not demonstrate significant improvement in the Satisfaction with Stroke Care Score (SASC-19) or QoL based on the 36-item Short-Form Health Survey (SF-36) after providing three telephone follow-up calls and one home visit from a stroke nurse [27]. Motivational Interviewing reported a trend towards improved self-reported medication adherence in the control group at 6 months which became a statistically significant change at 9 months [25]. However, all other measures were not significantly different, and given the difficulty of verifying medication adherence through self-report, the study was classified as not effective. As with several other studies, Motivation Interviewing observed a decrease in BP in both control and intervention groups; however, they did not differ from one another [25].

Table 1 provides definitions for and categories of study designs, target populations, intervention levels, delivery methods, study leaders, effectiveness, and study strategies.

## 4. Discussion

We learned several important lessons from both effective and non-effective studies in this review (Table 2). Based on the findings of studies with long-term effectiveness (SMART, DESERVE, NAILED, SPS3, INSPiRE-TMS, SRP), we observed that a successful program may utilize the exercise and physical rehabilitation principles of cardiac rehabilitation, BP control, and increased outpatient follow-up, specifically targeting medication titration [16,17,18,19,20,21]. Additionally, as demonstrated by the findings in NAILED and DESERVE, it will be important that an effective post-stroke care intervention includes a wide variety of tailored resources to suit different underserved groups from an education level and cultural standpoint [17,18].

*Targeting underserved high-risk groups*. NAILED showed effectiveness in targeting those with lower education levels, a group who may be traditionally underserved in traditional post-stroke care [18]. The program resulted in significant decreases in BP and LDL-C, regardless of education level, whereas the control group only saw improvements in the higher education groups, suggesting that the standard of care follow-up favors higher health literacy [18]. A clear link between health literacy levels and the ability to retain stroke education information has been identified, and given that smoking cessation, medication adherence, diet adjustment, and increased physical activity require patients to understand, retain, implement, and adhere to recommendations, it is important that tailored education can be offered [46,47]. In addition to low health literacy, several other groups remained underserved, and the racial/ethnic disparity in stroke outcomes is an ongoing public health challenge [48]. Minority groups are known to have more cardiovascular risk factors present at the time of index stroke [49]. Black and Hispanic patients have worse stroke outcomes compared to white patients [50]. There is a clear need for culturally tailored post-stroke care and DESERVE, a successful live intervention study, observed a nearly 10 mmHg drop in systolic BP in a subset of patients who identified as Hispanic, despite the overall group (all ethnicities included) not differing from controls [17]. Elements of their program, combined with culturally tailored initiatives such as TASHE (culturally tailored stroke education videos) [51] may be useful in providing traditionally underserved groups with effective stroke care [51].

*Controlling vascular risk factors*. One of the most important health targets following stroke is systolic BP, and the SPS3 trial looked at using a target of <130 mmHg in post-stroke patients (versus < 140 mmHg) with 3-monthly outpatient visits to adjust medications appropriately. They observed a downtrend in percent recurrent and no adverse effects associated with targeting a lower systolic BP, suggesting more rigorous systolic BP targets may be an important element of post-stroke care [19]. A large observational study identified that systolic BP values in the very low (<120 mmHg), high (140–150 mmHg), and very high (>150 mmHg) were associated with the risk of recurrent stroke [52] indicating somewhere between 120 and 140 mmHg would be the safest BP target. A large systematic review, looking at the combined outcomes of stroke, myocardial infarction, heart failure, cardiovascular death, and all-cause mortality, identified targets of <130 mmHg resulted in the best balance between safety and efficacy [53]. That being said, Mant et al. did not find clinically relevant decreases in blood pressure when using <130 mmHg as a target, compared to <140 mmHg as a target [54]. It is worth noting that SPS3 focused on patients with lacunar strokes specifically, which often result from small vessel atherosclerotic disease, a stroke etiology that is particularly linked to poorly controlled blood pressure [55].

Our study showed that interventions focusing on ensuring medications were prescribed, adhered to, and given at effective doses [14,18] seemed to be more successful than those that did not focus on medications [25,27,28,34,35,36,37,38]. However, some successful programs did not focus on medication [15,32,41,45] and some unsuccessful programs did [6,26,56]. That being said, counseling patients and providing follow-ups may not be effective if there is no opportunity to intervene when the current care plan is not working. NAILED, INSPiRE-TMS, and PREVENT incorporated the management of critical deviations from target values in their protocols [18,20,28]. NAILED and INSPiRE-TMS successfully demonstrated reductions in BP after stroke, whereas similar trials without built-in medication titration (DESERVE, PINGS, Aerobic Rehab, THRIVES, PRAISE, Adie et al. Feldman et al. and Motivational Interviewing) did not observe clinically relevant decreases in BP [17,18,20,22,24,25,30,31,40,42]. Interestingly the reduction in BP observed in INSPiRE-TMS did not translate into a significant difference in vascular events over three years, suggesting that while the study may provide an effective framework, BP and LDL-C targets may not have been rigorous enough [20]. INSPiRE-TMS and PREVENT offered medication titration via referral to the ED and PCP, versus direct titration as part of the program protocol (as seen in NAILED), and this may have contributed to the lack of observed reduction in cardiovascular events and BP, respectively [18,20,28]. Furthermore PROTECT, the Comprehensive Reminder System, Denny et al. and Kim et al. focused on standardizing pre-discharge care and showed that a proper care plan before the discharge is an essential element of an effective program [14,32,43,45,57].

*Choosing the right environment for delivering the program*. Programs that take place live require more staff and resources, hence from a cost-benefit standpoint, it is important that the methods being utilized are proven effective. SRP, for example, utilized a modified cardiac rehabilitation program for stroke patients [21]. The theory was that stroke and cardiovascular events share atherosclerotic pathophysiology and can both benefit from the same risk factor management regimens [21]. The foundation of cardiovascular rehab is reducing BP and blood sugar, getting active through low-impact exercise with a therapist, eating a healthier diet, losing weight, and smoking cessation [21]. SRP reported a hazard ratio of 0.1 (95% CI 0.01 to 0.9, *p* = 0.039) suggesting that controls had a nine times higher risk of death in the post-stroke period compared to participants. There was also a 1.5% 1-year unadjusted mortality rate in the participant group [21] which is significantly less than the 31% 1-year post-stroke unadjusted mortality rate identified in the general post-stroke population [58] Masterstroke and Aerobic Rehab also offered supervised exercise sessions and demonstrated improvement in cardiovascular health [15,42]. Aerobic Rehab was able to demonstrate significant differences in 6MWT and Met-minutes, despite 78% of the control group engaging in non-supervised exercise, suggesting that supervised exercise is superior, even in the face of improved health behaviors of the controls [42]. However, one must keep in mind that other initiatives with in-person exercise components were not nearly as successful, including LAST [39], ExStroke [38], Ticaa’dom [37] and the High-Intensive Exercise Program [36,37,38,39]. Keeping the supervised exercise regime close to a cardiac rehab may maximize the value of the intervention. Cardiac rehab programs are well established [59] and may be more accessible than stroke-specific rehabilitation programs. Programs that invested in the significant in-person follow-up, but were not centered around supervised exercise, were typically not as successful (Ticaa’dom, PRAISE, PREVENT, Outreach Nursing, and Feldman et al.) [24,27,28,37,60]. Furthermore, lengthy post-discharge education tailored in-person care plans can be costly and inconvenient for implementation [61]. The Comprehensive Reminder System was unique in that, after discharge education, they utilized automated messages, telephone calls, and written materials as a follow-up: all of which are relatively low resource-intensive options [43]. The latter can be compared to Ticaa’dom, who provided weekly phone calls, individual physical activity coaching, and home visits. Despite Ticaa’dom’s high one-on-one provider–client time, it was not more effective than the standard of care [37]. Telemedicine Guided Education [62], StrokeCoach [63], CEOPS [64] and a Structured Patient-Centered Educational Exchange [65] are all examples of ongoing research that utilize telephone follow-ups, which will potentially offer further insight into the efficacy of lower resource-intensive strategies.

Moving forward it is important that the social context and environment, which laid the groundwork for the index stroke or TIA, are identified, and addressed in secondary stroke prevention programs. The pre-intervention socio-economic determinants of health likely play a larger role in preventing secondary strokes than the actual intervention itself [66,67]. Feldman et al. utilized at-home nurse practitioner visits with the goal of addressing home behaviors that may cause barriers to successful rehabilitation; however, this intervention was not successful, suggesting that perhaps a broader social context, outside of the home, should be considered when designing future programs [24].

*Providing care in low resource areas*. An important element of reducing recurrent stroke is ensuring accessibility to care, even in lower resource settings [68,69,70]. PINGS looked at providing post-stroke care in Ghana and provided patients with pillboxes, BP monitors, and loaned cell phones, where they recorded their health data and received automated text message reminders [40]. The latter was a pilot study and not powered to detect a significant difference, but has demonstrated a trend towards an increasing proportion of participants meeting the BP target of <140/90 and improved medication possession rates. They highlighted the successful use of automated text messages, as a simple, low-cost alternative to in-person follow-up care, which could be particularly important for patients who cannot access specialized centers. BRIDGE-Stroke, a Level 2 intervention delivered in Latin America, was unfortunately unsuccessful in improving stroke outcomes; however, they highlighted the feasibility of delivering training workshops, treatment algorithm reminders, and training materials to physicians in lower resource settings [23]. Further work on how to translate Level 2 initiatives into successful outcomes in low resource settings will need to be established, as lower and middle-income countries are typically more burdened by recurrent strokes [70]. Increasing the use of supervised activity programs is particularly challenging in rural or geographically isolated settings; however, housing interventions in the community-based multi-use spaces or providing patients with virtual supervision may help decrease barriers to accessing this care in the future [71,72]. The SPRITE trial (ongoing) will assess the efficacy of a home-based cardiac rehabilitation program, which may further increase accessibility. Exploring the use of apps, wearable devices (i.e., FitBit or Apple watches), and text reminders in combination with other initiatives will be an important future direction and may add an additional dimension of care, particularly for those who cannot attend frequent in-person visits.

*Improving the transition of care*. Assisting patients as they navigate through a variety of healthcare settings, the ED, stroke ward, rehabilitation center, outpatient, primary care, and eventually home, is essential to preventing recurrent strokes [73,74]. Very few studies in this review focused on the transition of care (TOC), but it is a well-established element of successful rehabilitation programs in heart failure [75]. However, the high acuity nature and busy environment of the stroke ward, compared to that of more insidious onset diseases such as heart failure, may lead to poorer handover between care settings and decrease the efficacy of TOC [76]. A heart failure care study ascertained that while TOC programs are more costly than the standard of care, they are also more effective, and advocated for nurse home visits [77]. Another study described the importance of increasing the ease of referral, which is an essential component of TOC. They specifically looked at referring obese patients to weight loss programs, but the same referral principles can be applied to post-stroke care [78]. The use of electronic reminders for physicians and “single-click” referral through an EHR work-flow were promising tools; however, when the Point of Care EHR Prompt study similarly attempted to flag patients for secondary prevention based on risk factors, there was a very low uptake by physicians and the intervention was ultimately not efficacious [26,78]. Perhaps the EHR-based tools need to be refined with input from providers in future program development.

A large review on the efficacy of post-MI and post-stroke TOC services was inconclusive in its findings and was limited by the lack of clarity on what standard of care is after stroke with regards to TOC [79]. So far, home visits have not been proven efficacious in post-stroke care, but it is an area that needs further investigation before conclusions can be drawn [27,37]. A pharmacist-driven TOC clinic trial in Memphis, Tennessee, offered education, medication titration, and referral to primary care services to uninsured stroke patients [80]. Despite only offering patients a once-off clinic appointment, Nathans et al. observed a significant reduction in 90-day hospital readmission rates. Some degree of focus on TOC is likely necessary to make an effective program; however, the manner in which it is best delivered has yet to be elucidated.

*Study limitations and future directions*. The search strategy did not generate as many titles as we anticipated and there may have been some studies that were inadvertently screened out by the limitation placed on each search engine, this is further evidenced by the complete lack of Level 3 and 4 intervention studies obtained. As well, we were unable to provide a numerical or statistical comparison of studies to support the conclusions we drew. Directly comparing studies in this review was not possible given the vastly different populations, outcome measures, and analysis conducted by each individual study. While observing trends can be helpful for discussion purposes, this may not be able to fully answer the following question: what exactly constitutes a successful recurrent stroke prevention program? Moving forward, it would be beneficial if some degree of standardization for measuring the success of stroke recovery programs was introduced, to allow for meta-analyses to be conducted in the future. Given that we did not assess raw data in this review, we did not assess for power or risk of bias, which may have limited our ability to accurately categorize studies as effective or not effective. Furthermore, several studies are still recruiting or their RCT results are not yet published; therefore, we were unable to include this information in our review. Only English texts were included which may have made it appear that fewer studies were being conducted in Africa, Latin America, Asia, and the Middle East. Only one study was conducted cross-continentally, which limits our ability to compare efficacy by region. Finally, the need for multi-level interventions is clear, but unfortunately, data in this area are still lacking. Finding out how to combine interventions targeting individuals, physicians, health systems, and communities is not something we can comment on in this review but is an essential next step in this field of work [81].

Table 2 highlights current evidence for our recommendations outlined in the discussion section.

**Table 2:** Summary of Lessons Learned.

Lesson	Evidence
1. Offer tailored care for different ethnic/minority groups and those with low health literacy.	Boden-Albala et al. [17]
	Irewall et al. [18]
	Sanders et al. [46]
	Rolls et al. [47]
	Ravenell et al. [51]
2. Aim for tight blood pressure control.	Ovbiagele et al. [10]
	SPS3 Investigators [19]
	Bangalore et al. [53]
3. Provide ample opportunities to titrate medications.	Boden-Albala et al. [17]
	Irewall et al. [18]
	Ahmadi et al. [20]
	Adie et al. [22]
	Feldman et al. [24]
	Barker-Collo et al. [25]
	Hornnes et al. [28]
	Owolabi et al. [31]
	Sarfo et al. [40]
	Toledano-Zarhl et al. [42]
	Kronish et al. [60]
4. Establish and implement the care plan prior to discharge through standardized education and prescriptions.	Ovbiagele et al. [10]
	Denny et al. [32]
	Wan et al. [41,43]
	Kim et al. [45]
	Benoit et al. [61]
5. Invest in supervised exercise and low resource-intensive (telephone/automated/written materials) follow-up, but do not invest in other forms of in-person intervention (i.e., nurse home visits, repeated in-person counseling/education sessions).	Cuccurullo et al. [21]
	Wan et al. [41,43]
	Toledano-Zarhl et al. [42]
	White et al. [50]
	Benoit et al. [61]
6. Remove possible barriers to accessibility of care, particularly in lower resource settings.	Sarfo et al. [40]
	Machline-Carrion et al. [56]
	Smith et al. [68]
	Urimubenshi et al. [69]
	Pandian et al. [70]
7. Improve transition of post-stroke care throughout various healthcare settings.	Cameron et al. [73]
	Broderick et al. [74]
	Rattray et al. [76]
	Blum et al. [77]
	Bettger et al. [79]
	Nathans et al. [80]

Table 3 summarizes the relevant findings of each study which led to their classification as long-term effective, short-term effective, or not effective.

**Table 3:** Summary of Intervention Effectiveness and Study Results.

Intervention	Short Term (<6 Months)	Long Term (≥6 Months)
Comprehensive Reminder System [41]	Effective at 3-months post-randomization HLPL II score 3.16 in IG and 2.79 in CG Increased PA ^1^ in IG Improved nutrition in IG Reduced salt intake in IG Improved medication adherence in IG A 1.38 mmHg decrease in BP in CG vs. 9.86 mmHg decrease in IG A 1.38 mmHg decrease in BP ^1^ in CG vs. 9.86 mmHg decrease in IG No difference observed in smoking or alcohol use	No assessment after 6 months
PROTECT [52]	Effective immediately/at the time of discharge Increase in the number of patients prescribed a statin, ACEi/ARB ^1^ or thiazide diuretics, but no difference in antithrombotic therapy initiation A total of 100% patient education achieved before discharge No data on the longevity of change	No assessment after 6 months
SRP [21]	Effective at 120-days post-randomization AM-PAC ^1^ mobility and daily activity score improved, but no difference was observed in the applied cognitive score Increase in MET-minutes ^1^ A total of 26 of 136 IG participants ended early due to CV ^1^ complications	Effective at 1-year post-randomization in terms of 1-year mortality IG 1-year unadjusted mortality of 1.47% CG 1-year unadjusted mortality of 31.1% Nonparticipants have a 9.09× higher chance of dying than participants Note: follow-up range was 1 day–1 year (median = 85 days); therefore, the classification of “long-term” effectiveness may not be accurate
Masterstroke [15]	Effective immediately and at 3-months post-program conclusion Reduced salt intake Improved fat and fiber barometer score Improved TUG ^1^ test Improved stroke knowledge (score not validated) Improved QoL ^1^ In qualitative analysis (*n* = 9) participants reported perceived benefit of exercise No difference in 6MWT ^1^, resting heart rate, and waist circumference	No assessment after 6 months; however, most participants continued to use Masterstroke gym for exercise after the conclusion of the study
NAILED [18]	No assessment before 6 months	Effective at 12-months post-randomization or discharge IG showed a favorable change in SBP ^1^ regardless of education level IG showed a more favorable change in LDL-C ^1^ in the lower education group CG (standard of care) favored higher education groups in terms of improved SBP and LDL-C
Kim et al. [45]	Effective at 3-months post-randomization Increased exercise Reduced salty food intake Increased fruit and vegetable intake Increased sense of control Increased health motivation Improved caregiver mastery No difference in lipids, smoking, drinking, or med adherence Deemed to be feasible Small sample size and low power may have limited the findings	No assessment after 6 months
Aerobic Rehab [42]	Effective at 6-weeks post-randomization Increased exercise 6MWT ^1^ Increased FSST ^1^ Increased METs No difference in stairs, resting HR ^1^, or blood pressure Small sample size and high participant dropout rate A total of 78% of CG performed exercise protocol daily, making it difficult to establish significant IG vs. CG differences	No assessment after 6 months
PINGS [40]	Effective at 3-months post-randomization Better medication possession ratio in IG vs. CG Improved autonomous regulation scores from baseline to 3 months in both CG and IG Significant improvement in confidence in taking medications as prescribed in IG, not seen in CG No difference in proportion reaching BP of <140/90, but trending towards significance Note: these are interim results of the pilot study only; however, preliminary results were reported with a control and intervention pilot group. Protocol dictates follow-up at 9 months and the current pilot study was not powered to detect a significant difference.	No assessment after 6 months
DESERVE [17]	No assessment before 6 months	Effective at 12-months post-randomization (in Hispanic patients only) Drop in BP at 12 months in Hispanic IG group compared to Hispanic CG No difference in overall group SBP Note: the study was not powered for this sub-analysis
SMART [16]	Effective at 6-months post-randomization Statin adherence of 59% in IG compared to 37.2% adherence in CG at 6 months No difference between CG and IG in antiplatelet, antihypertensive, anti-diabetic, or anticoagulant adherence at 6 months No difference between CG and IG in terms of the secondary composite outcome	Effective at 12-months post-randomization Statin adherence of 56% in IG compared to 33% adherence in CG at 12 months No difference between CG and IG in antiplatelet, antihypertensive, anti-diabetic, or anticoagulant adherence at 12 months No difference between CG and IG in terms of the secondary composite outcome
INSPiRE-TMS [20]	No assessment before 6 months	Effective at 3-years post-randomization Improved BP, LDL-C, PA, smoking abstinence, antiplatelet use, HbA1c, change in BMI, and stair-climbing test in IG at 1 year ^1^ BP, LDL, PA, smoking abstinence, and stair-climbing test improvements were maintained at 2 years in IG BP, LDL, PA, and stair-climbing test improvements were maintained at 3 years in IG Results did not translate to decreased vascular events
SPS3 [19]	No assessment before 6 months	Effective at 1-year post-randomization Reduced intracerebral hemorrhagic stroke rates in IG compared to CG Non-statistically significant trend toward reduction in all strokes Limited side effects associated with IG target BP vs. CG target BP
Denny et al. [32]	Not Effective at 30-days post-discharge Increased knowledge score following educational video; however, knowledge score was not validated and level of improvement (only 1 point) has limited clinical significance	No assessment after 6 months
SEP [33]	Not Effective at 6-months post-randomization Improved social functioning sub-score of the SF-36 ^1^ in CG caregivers vs. IG caregivers	No assessment after 6 months
THRIVES [31]	Not Effective at 1-,3- and 6-months post-randomization Reduced BP in CG and IG at 1, 3, and 6 months, but no observed difference between groups	Not Effective at 9- and 12-months post-randomization Reduced BP in CG and IG at 9 and 12 months, but no observed difference between groups
PRAISE [60]	No assessment before 6 months	Not Effective at 6-months post-randomization The magnitude of SBP reduction was not clinically significant No difference in the composite outcome, LDL-C, antithrombotic use, medication adherence, DBP ^1^, or proportion with depressive symptoms
LAST [39]	No assessment before 6 months	Not Effective at 18-months post-randomization No difference in MAS ^1^ or secondary outcomes Improved TUG in CG
Adie et al. [22]	Not Effective at 6-months post-randomization No difference in ambulatory SBP or secondary outcomes, except for improved medication knowledge in IG (*p* < 0.001); however, increased medication knowledge did not result in improved adherence or reduced BP Total cholesterol levels decreased, and statin use increased in all groups; however, there was no difference between IG and CG	No assessment after 6 months
ExStroke [38]	No assessment before 6 months	Not Effective at 24-months post-randomization No difference in PASE ^1^ score at 24 months IG participants who attended all visits had a higher PASE score than controls but this was no longer significant after the pre-intervention PASE score was controlled for in the analysis
Feldman et al. [24].	Not Effective at 3-months post-randomization All three groups had decreased BP from baseline, but IG and CG groups were not different from each other	Not Effective at 12-months post-randomization All three groups had decreased BP from baseline, but IG and CG groups were not different from each other
Ticaa’dom [37]	Not Effective at discharge and 6-months post-randomization Increased 6MWT distance was observed in IG from baseline to 6 months, increased walk distance not observed in CG; however, 6MWT results were not statistically different between IG and CG Improved mFAC ^1^ in IG, but not CG, at 6 months No difference in other secondary outcomes	Not Effective at 12-months post-randomization Increased 6MWT distance was observed in IG from baseline to 6 months, increased walk distance not observed in CG; however, 6MWT results were not statistically different between IG and CG Improved mFAC at 6 months was not maintained at 12 months No difference in other secondary outcomes
Point of Care EHR Prompt [26]	No assessment before 6 months	Not Effective at 15-months post-randomization No difference in any measures Note: 25% of IG practices did not access the software
High-Intensive Exercise Program [36]	Not Effective at 3- and 6-months post-randomization No difference in SF-36 or GDS ^1^ at 3 or 6 months Improved mental component and mental health subscales of SF-36 in the CG at 3 months	No assessment after 6 months
PREVENT [28]	No assessment before 6 months	Not Effective at 12-months post-randomization No difference in any outcomes, except for slightly lower DBP in IG after the intervention, compared to CG
STARS-Plus [6]	No assessment before 6 months	Not Effective at 12-months post-randomization No difference in 30-day and 365-day SF-12 ^1^ score All participants who responded at each follow up self-reported improved medication adherence; however, there was no statistically significant correlation to their SF-12 score
DMP [29]	No assessment before 6 months	Not Effective at 30-months post-randomization No difference in any measures Post hoc analysis Forest plot favored DMP in terms of all vascular events in pt > 65 years, with no chronic kidney disease, and who were non-smokers Study was underpowered
ICMP [34]	Not Effective at 1- and 12-weeks post-intervention completion No significant difference in any measures Note: 10% dropout rate	No assessment after 6 months
Education and Support Package [35]	Not Effective at 3-months post-randomization No difference in stroke knowledge, anxiety, depression, quality of life, or caregiver strain IG had significantly better self-efficacy in accessing stroke information, feeling informed and satisfaction with information received, but each category was only represented by one item on a non-validated scale	No assessment after 6 months
BRIDGE Stroke [56]	Not Effective at 90-days post-randomization No difference in the composite outcome No difference in secondary outcomes except for more patients in IG had a tPA ^1^ door-to-needle time of <45 min IG had a higher rate of hemorrhagic transformation than CG The feasibility of working in lower resource settings utilizing a Level 2 intervention was successfully demonstrated	No assessment after 6 months
Outreach Nursing [27]	Not Effective at 6-months post-discharge No difference in any measures, except for reduced use of rehab and reduced anxiety scores were observed in IG group	No assessment after 6 months
Motivational Interviewing [25]	Not Effective at 3- and 6-months post-randomization No difference in any measures A trend towards increased self-reported medication adherence at 6 months in IG	Not Effective at 9- and 12-months post-randomization No difference in any measures except for increased self-reported medication adherence at 9 months in IG There was a significant decrease in BP seen in IG and CG, but no difference between groups

^1^ Abbreviations include Health-Promoting Lifestyle Profile II Score (HLPL), intervention group (IG), control group (CG), blood pressure (BP), systolic blood pressure (SBP), diastolic blood pressure (DBP), heart rate (HR), low-density lipoprotein cholesterol (LDL-C), angiotensin-converting enzyme inhibitors (ACEi), angiotensin receptor blocker (ARB), cardiovascular (CV), Activity Measurement for Post-Acute Care (AM-PAC), metabolic equivalents (METs), hazard ratio (HR), Timed Up and Go Test (TUG), quality of life (QoL), 6-Minute Walk Test (6MWT), Four Square Step Test (FSST), physical activity (PA), risk factor (RF), patient-year (pt-yr), 36-item Short Form Survey (SF-36), 12-item Short Form Survey (SF-12), Motor Assessment Scale (MAS), Physical Activity Scale for the Elderly (PASE), Geriatric Depression Scale (GDS), Analysis of Variance (ANOVA), and tissue plasminogen activator (tPA).

Table 4 provides a summary of intervention titles, year of publication, first author, sample size, study design, country of origin, intervention target population, level of intervention, setting of the intervention, provider of the intervention, and goal of the intervention for all studies included in the review.

**Table 4:** Summary of Basic Study Information.

Intervention	Author	Yr ^1^	N ^1^	Design	Location	Target	Level	Delivery	Leader	Strategy
Comprehensive Reminder System	Wan et al. [41]	2018	174	RCT ^1^	China	Patient	1	Live Written materials Automated	Nurse	Education Adherence
PROTECT	Ovbiagele et al. [52]	2004	130	Non-RCT	USA	Provider	2	Live Written materials	HCW ^1^	Adherence
Masterstroke	White et al. [15]	2013	22	Mixed methods	Australia	Patient	1	Live	MDT ^1^	Education PA
NAILED	Irewall et al. [18]	2019	771	RCT	Sweden	Patient	1	Live	Nurse	Adherence
*n/a*	Kim et al. [45]	2013	102	RCT	South Korea	Patient Caregiver	1	Virtual	Researcher	Education Adherence
Aerobic Rehab	Toledano-Zarhl et al. [42]	2011	28	RCT	Middle East	Patient	1	Live	AHP ^1^	PA
PINGS	Sarfo et al. [40]	2019	60	RCT	Ghana	Patient	1	Live Automated	Nurse	Adherence
DESERVE	Boden-Albala et al. [17]	2019	552	RCT	USA	Patient	1	Virtual Written materials Live	Care Coordinator	Adherence
SMART	Peng et al. [16]	2014	3821	Cluster RCT	China	Patient	1	Live Virtual	HCW	Adherence Education
INSPiRE-TMS	Ahmadi et al. [20]	2020	2098	RCT	Germany	Patient	1	Live	HCW	Adherence
SPS3	SPS3 Investigator Group [19]	2013	3020	RCT	USA	Patient	1	Live	Physician	Adherence
n/a	Denny et al. [32]	2017	93	Pre- and post-test	USA	Patient	1	Virtual	HCW	Education
SEP	Rodgers et al. [33]	1999	204	RCT	UK	Patient Caregiver	1	Live	MDT	Education
THRIVES	Owolabi et al. [31]	2019	400	RCT	Nigeria	Patient	1	Automated Virtual Written materials	Physician	Adherence Education
PRAISE	Kronish et al. [60]	2013	600	RCT	USA	Patient	1	Live Written materials	Peer group leader	Adherence Education
LAST	Askim et al. [39]	2018	380	RCT	Norway	Patient	1	Live	AHP	PA
n/a	Adie et al. [22]	2010	56	RCT	UK	Patient	1	Live	Researcher	Adherence
ExStroke	Boysen et al. [38]	2009	314	RCT	Denmark China Poland Estonia	Patient	1	Live	HCW	PA
n/a	Feldman et al. [24]	2020	495	3 arm RCT	USA	Patient	1	Live	Nurse practitioner	Adherence
Ticaa’dom	Mandigout et al. [37]	2020	83	RCT	France	Patient	1	Live Virtual	AHP	PA
Point of Care EHR Prompt	Dregan et al. [26]	2014	106 sites, 11391 pt	Cluster RCT	UK	Provider	2	Virtual Written materials	Computer-led	Adherence
High-Intensive Exercise Program	Holmgren et al. [36]	2010	34	RCT	Sweden	Patient	1	Live	MDT	PA Education
PREVENT	Hornnes et al. [28]	2011	344	RCT	Denmark	Patient	1	Live	Nurse	Adherence
STARS-Plus	Bretz et al. [6]	2014	193	Pre- and post-test	USA	Patient	1	Live	HCW	Education Adherence
DMP	Fukuoka et al. [29]	2019	321	RCT	Japan	Patient	1	Written materials	Nurse	Education Adherence
ICMP	Maasland et al. [34]	2007	65	RCT	Netherlands	Patient	1	Virtual	Physician	Education
Education and Support Package	Eames et al. [35]	2012	119	RCT	Australia	Patient Caregiver	1	Live Written materials	AHP	Education
BRIDGE Stroke	Machline-Carrion et al. [56]	2019	36 sites, 1642 pt	Cluster RCT	Latin America	Provider	1	Live Written materials	Care coordinator	Education Adherence
Outreach Nursing	Boter et al. [27]	2004	536	RCT	Netherlands	Patient Caregiver	1	Live	Nurse	Adherence
Motivational Interviewing	Barker-Collo et al. [25]	2015	386	RCT	New Zealand	Patient	1	Live	Researcher	Adherence

^1^ Abbreviations include the year of publication (Yr), sample size (N), randomized control trial (RCT), patients (pt), healthcare worker (HCW), multidisciplinary team (MDT), allied health professional (AHP), and physical activity (PA).

## 5. Conclusions

Building a gold-standard stroke rehabilitation program with the aim of reducing the rate of recurrent ischemic strokes is challenging and there is not necessarily one correct strategy. One should aim to offer a simple and accessible regime that begins in the hospital with education and correct prescriptions on discharge, followed up with long-term tight BP control, a supervised exercise program (modeled after post-cardiac event rehabilitation), opportunities for medication titration if goals are not met, telephone or text message follow-ups, and improved transition of care after discharge.

## Figures and Tables

**Table 1:** Summary of Study Definitions and Categories.

Study Design	Target Population	Level of Intervention ^1^	Delivery Method ^1^	Study Leader ^1^	Effectiveness	Strategy
What was the outlined design strategy referenced in the “methods” section?	Who was the study defined to benefit?	Where in the healthcare model was the intervention targeted to take effect?	How was the intervention provided to the target group?	Who oversaw the delivery of the intervention to the target group?	Was the study effectively able to accomplish significant change compared to the control group or pre-test levels?	What is the overarching aim of the intervention?
RCT ^2^ Non-RCT ^2^ Cluster randomized, Prospective cohort, Mixed methods, Pre/post-test	Patients Caregivers Providers	One (individual) Two (provider)	Live Written materials Automated Virtual	Nurse Physician Researcher HCW ^2^ MDT ^2^ AHP ^2^ Care coordinator Peer Leader Computer-led	Long-term Effective Short-term Effective Not Effective	Adherence to Care Plan Education Physical Activity

^1^ Intervention level was defined as ‘Level One’ (individual improvement of health behavior), ‘Level Two’ (empowering healthcare providers to deliver better care), ‘Level Three’ (promotion of healthy behaviors at a community level), or ‘Level Four’ (policy-level change). Only studies with Level One and Two interventions were obtained for this review using the predefined search strategy. The delivery method was defined as either ‘live’, ‘written materials’, ‘automated’, or ‘virtual’. ‘Live’ strategies occurred over the phone or in-person. In-person initiatives occurred in the patient’s home or outside the home (i.e., in the hospital, outpatient, gym, or community center). ‘Written materials’ included trials that offered handbooks, calendars, information leaflets, workbooks, patient report cards, journals, posters, and printed reminders. ‘Automated’ strategies included text messages or automated voice recording reminders. ‘Virtual’ strategies included video/audio material, interactive websites and web tools, activity monitors, and EHR prompts. ‘Healthcare worker (HCW)’ refers to anyone from the healthcare team, including doctors and nurses, not further specified in the methods. ‘Multidisciplinary team (MDT)’ refers to anyone from the healthcare or research team involved in patient care or intervention delivery, not further specified in the methods. ‘Computer-led’ refers to programs, or program elements, that run entirely electronically and do not require a person to deliver or supervise the delivery of the intervention. ^2^ Abbreviations include randomized control trial (RCT), healthcare worker (HCW), multidisciplinary team (MDT), and allied health professional (AHP).

## Data Availability

Not applicable.

## Supplementary Materials

The following are available online at https://www.mdpi.com/article/10.3390/jcm10184209/s1, Figure S1: PRISMA Flow Chart. Table S1: Intervention Description and Primary Outcome measures/Measurement Methods. Table S2: Ongoing, pilot, and feasibility study summary. Table S3: PRISMA Checklist.
