# Supplementary material for: “Lessons Learned” Preventing Recurrent Ischemic Strokes through Secondary Prevention Programs: A Systematic Review
Source: J Clin Med. 2021 Sep 17;10(18):4209. doi: 10.3390/jcm10184209 (PMC8471819; doi:10.3390/jcm10184209)
Supplement: Supplementary file 1 [file jcm-10-04209-s001.zip › jcm-1315235-supplementary.pdf]

“Lessons Learned” Preventing recurrent ischemic strokes through secondary prevention program: A systematic review

Supplemental Materials

Table S1. Intervention Description and Primary Outcome Measures / Measurement Methods

Intervention	**Delivery Method and Study Strategy**<br>*(IG = intervention group, CG = Control Group)*	**Primary Outcome Measures / Measurement Methods**
Wan et al. (Comprehensive Reminder System)	**IG:** Face-to-face education before discharge, patient calendar handbook (assessing disease risk, assessing deviation from guidelines, establishing health belief, imparting knowledge and skills, goal setting), telephone health belief education, and discussion of barriers to implementing health behavior and automated SMS (reminders for clinic visits, lifestyle modifications, med adherence, health belief, and precautions)<br><br>**CG:** standard of care	**Primary Outcome Goal:** Improved education and adherence to care plan<br><br>**Measurement Method:**<br>1. HLPL II score at baseline and 3 months<br>2. HLPL II subcategory scores at baseline and 3 months (PA, nutrition, low salt diet, smoking, alcohol, medication adherence)<br>3. Blood pressure at baseline and 3 months
Ovbiagele et al. (PROTECT)	**IG:** Implementation of antithrombotic, statins, ACEi/ARBs, and thiazides as well as patient education on smoking cessation, AHA diet, exercise, and stroke warning signs pre-discharge, achieved through educational resources, training, and pre-printed admission orders for physicians + patient education leaflets<br>**CG:** 117 not-enrolled historical consecutive patients encountered in the previous year	**Primary Outcome Goal:** Improved adherence to care plan *at the physician level*<br><br>**Measurement Method:**<br>1. Initiation and prescription of antithrombotic, statin, ACEi/ARB, and thiazide before discharge<br>2. Patient education achieved, and written material provided before discharge
Denny et al.	**IG:** 5-minute educational stroke video about defining a stroke, stroke symptom recognition, stroke RF, stroke prevention, importance of OPD follow up and self-efficacy in calling 911, played at the bedside before discharge<br><br>**CG:** standard of care education only	**Primary Outcome Goal:** Improved education<br><br>**Measurement Method:** Investigator developed 10 item questionnaires immediately (knowledge acquisition) and at 30 days (knowledge retention)
Cuccurullo et al. (SRP)	**IG:** physician visits at 30, 60, 90, and 120 days after stroke, modified cardiac rehabilitation exercise program utilizing low-moderate level exercise (differences: group therapy, no inclusion of perceived dyspnea scale in modified)<br><br>**CG:** standard of care	**Primary Outcome Goal:** Improved exercise and adherence to the care plan<br><br>**Measurement Method:**<br>1. Cox proportional hazard ratio analysis of SRP vs. Get with the Guidelines stroke patients 1-year mortality rate<br>2. MET min (within-group, pre-post)<br>3. AM-PAC score (between groups)
White et al. (Masterstroke)	**IG:** 1-hour education (risk factors, nutrition, diet, and managing stroke complications) and 1-hour supervised exercise session at moderate level exercise, once per week, for 9 weeks<br><br>**CG:** *No control group*	**Primary Outcome Goal:** Improved education and exercise<br><br>**Measurement Method:**<br>1. The Stroke and Aphasia QoL Scale (SaQoL-39),

		2. Waist circumference, resting heart rate, TUG, 6MWT, Fat & Fiber Barometer, self-report daily salt intake<br>3. Investigator developed knowledge questionnaire
Irewall et al.<br>(NAILED)	**IG:** 1- and 12-month post-discharge telephone phone calls with a nurse to go over blood pressure and blood chemistry results, provide lifestyle counseling, and titrate medication<br><br>**CG:** Nurse phone calls without counseling or changes to treatment, BP and blood results were forwarded to GP	**Primary Outcome Goal:** Improved adherence to the care plan<br><br>**Measurement Method:** SBP at 1 and 12 months<br><br>**Secondary Outcomes:**<br>1. DBP at 12 months<br>2. LDL-C at 12 months
Kim et al.	**IG:** 9 sessions of web-based stroke education for patients and caregivers that offered repeatable video lectures with tips from HCW about stroke prevention (+automated quizzes), automatic feedback based on patients self-rated health behaviors, an optional email service network connecting patients with HCWs, and external links to stroke education websites.<br><br>**CG:** standard of care education only	**Primary Outcome Goal:** Improved education and adherence to care plan<br><br>**Measurement Method:** Blood lipids, self-reported health behavior (including exercise, smoking, alcohol, salty food, fruit and vegetable consumption, and medication adherence), Mastery Scale, Health Motivation Scale, and Care Giving Mastery Scale<br><br>**Secondary Outcome:** Feasibility
Toledano-Zarhl et al.<br>(Aerobic Rehab)	**IG:** Twice weekly 33-55 min of supervised treadmill/hand bike/stationary bike exercise at 50-70% max HR and once weekly 45-55 min group strength/flexibility/coordination sessions<br><br>**CG:** Provided with exercise protocol, but not supervised	**Primary Outcome Goal:** Improved exercise<br><br>**Measurement Method:** 6MWT, stair test (time to ascend and descend 13 stair flight), four square step test (FSST), BP, HR, and MET-mins at 1 and 6 weeks
Sarfo et al.<br>(PINGS)	**IG:** Pillbox and BP monitor provided to patients, reminder and reinforcement SMS messages regarding BP recording (every 3 days) and medication adherence (daily). Texts taper to the variable interval (average every 3 days) after 2 weeks of 100% adherence. Lack of adherence or missed BP measurements is followed up with motivational interviewing from a nurse. The intervention lasts for 3 months.<br><br>**CG:** receive SMS messages every 3 days with lifestyle behavior advice but no medication adherence or HTN/Stroke related SMS	**Primary Outcome Goal:** Improved adherence to the care plan<br><br>**Measurement Method:** Blood pressure<br><br>**Secondary Outcomes:** Medication adherence, (medication possession ratio), HTN management competence (18 items perceived confidence scale), autonomous self-regulation (15 item treatment self-regulation questionnaire)
Boden-Albala et al.<br>(DESERVE)	**IG:** education PowerPoint presentation, motivational video, and workbook received at discharge, 1 month follow up reminder and telephone communication about workbook and post-visit follow up call, 3- month telephone workbook review in addition to standard of care<br><br>**CG:** Standard of care	**Primary Outcome Goal:** improved adherence to the care plan<br><br>**Measurement Method:** SBP

Peng et al.<br>(SMART)	**IG:** Lifestyle modification (smoking cessation, healthy diet, exercise) and interactive education session before discharge + access to SMART website with risk factor and lifestyle change education<br><br>**CG:** standard of care	**Primary Outcome Goal:** Improved adherence to care plan and education<br><br>**Measurement Method:**<br>Adherence to antiplatelets, antihypertensives, anti-diabetic meds, anticoagulation meds, and statins<br><br>**Secondary Outcomes:** recurrent stroke, new hemorrhagic stroke, ACS, and all-cause mortality (composite outcome)
Ahmadi et al.<br>(INSPiRE-TMS)	**IG:** 8 outpatient 1-to-1 visits over 2 years focusing on motivational interviewing, feedback, plans for risk factor management, physical fitness assessment, access to complementary services (i.e., peer groups), and management of critical deviations of target values through the emergency department<br><br>**CG:** standard of care	**Primary Outcome Goal:** improved adherence to the care plan<br><br>**Measurement Method:** Composite major vascular event (secondary stroke, ACS, vascular death)<br><br>**Secondary Outcomes:** Stroke, ACS, vascular death non-vascular death, hospitalizations, level of dependency (mRS), days alive and at home, BMI, physical fitness level (stair-climbing test), and RF management (BP, LDL, HbA1c)
SPS3 Investigator Group<br>(SPS3)	**IG:** Seen in outpatient every month for 3 months and then quarterly for BP measurement and medication adjustment with a goal SBP of <130mmHg<br>**CG:** Seen in outpatient every month for 3 months and then quarterly for BP measurement and medication adjustment with a goal SBP of 130-149mmHg, if BP fell below 130 their BP medications were reduced or stopped	**Primary Outcome Goal:** Improved adherence to the care plan<br><br>**Measurement Method:** Recurrent stroke
Rodgers et al.<br>(SEP)	**IG:** 7 stroke education sessions for survivors and their careers; 1 session as inpatient and 6 as an outpatient, covering the nature of stroke, the psychological effect of stroke, the role of OT and PT, caring for someone who has suffered a stroke, communication/swallowing problems after stroke, reducing the risk of stroke<br><br>**Control:** standard of care	**Primary Outcome Goal:** Improved education<br><br>**Measurement Method:** Patient and caregiver perceived health status (SF-36)<br><br>**Secondary Outcomes:** Investigator developed stroke knowledge questionnaire, satisfaction with services, Hospital Anxiety and Depression scale (patients) and General Health Questionnaire (careers), disability (Nottingham E-ADL and Oxford Handicap scale)
Owolabi et al.<br>(THRIVES)	**IG:** pre-appointment text reminders, in clinical education videos, a patient report card on patients' current control of key RFs, and post clinic text to emphasize better RF control<br><br>**CG:** standard of care only + text message study updates	**Primary Outcome Goal:** Improved education and adherence to care plan<br><br>**Measurement Method:** SBP at 1, 3, 6, 9, and 12 months
Kronish et al.<br>(PRAISE)	**IG:** peer-led workshops for 6 weeks (90 min sessions once weekly) focusing on the biology of stroke, stroke treatments, the importance of adherence to preventative measures, optimizing medication adherence, formation of action plans (weekly), feedback on progress, and culturally sensitive education packet<br><br>**CG:** waitlist group (received access to resources after 1 year of wait time)	**Primary Outcome Goal:** Improved education and adherence to care plan<br><br>**Measurement Method:** BP and LDL-C and use of antithrombotic medications (composite outcome)<br><br>**Secondary Outcomes:** BP, LDL-C, use of antithrombotic, pre-post BP changes, pre-post LDL-C changes, pre-post self-reported medication adherence, the proportion of participants with elevated depression signs and symptoms
Askim et al.<br>(LAST)	**IG:** standard of care + 18 months of individualized coaching by PT (including 45-60 min/week of supervised vigorous exercise along with 30 mins of individual exercise	**Primary Outcome Goal:** Improved exercise

	7 days a week), a training diary, and access to community treatment groups. The first 6 meetings were face-to-face, the next 6 meetings were alternating face-to-face, and phone and the final 6 meetings were 4 phone calls and 2 face-to-face meetings<br><br>**CG:** standard of care in Norway (stroke unit care, rehab, 3-month follow-up appointment, 45 min of physiotherapy per week at patients' home for 3-6 months depending on stroke severity)	**Measurement Method:** Motor Assessment Scale<br><br>**Secondary Outcomes:** Independence (Barthel index and mRS), balance (Berg Balance Scale Item 14 and TUG), walking ability (10 min max gait speed and 6MWT), QoL (Stroke Impact Scale 3.0, EQ-5D-5L), fatigue (fatigue severity scale), hospital anxiety and depression scale, MMSE, caregiver strain index
Adie et al.	**IG:** 4x 20-minute telephone follow up sessions with brief motivational intervention based on social cognitive theory + medication, BP, smoking, diet, and exercise review+ goal setting at 7-10 days, 1, 2, and 4 months<br><br>**CG:** standard of care	**Primary Outcome Goal:** Improved adherence to the care plan<br><br>**Measurement Method:** 12-hour ambulatory SBP change<br><br>**Secondary Outcomes:** Antihypertensive and cholesterol-lowering agent use, random total cholesterol, self-reported exercise, number of health care contacts, smoking status, and medication knowledge
Boysen et al. (ExStroke)	**IG:** Received detailed individualized physical activity training plan at discharge, follow-up visits with an instructor to discuss physical activity plan and make an" agreement" occurred at 3, 6, 9, 12, 18, and 24 months. In between visits, there were phone calls to discuss adherence to the agreement.<br><br>**CG:** Follow up visits at 3, 6, 9, 12, 18, and 24 months with no physical activity focus	**Primary Outcome Goal:** Improved exercise<br><br>**Measurement Method:** Physical Activity Scale for the Elderly score<br><br>**Secondary Outcomes:** time from randomization to recurrent stroke, MI, death from any cause, vascular death, frequency of recurrent stroke, mRS, falls and fractures
Feldman et al.	**IG 1:** usual home care + 30-day transitional NP visiting 3 times in the home and 3 times on the telephone to do a comprehensive health assessment, monitor BP, ensure medication and behavioral regimes are followed, collaborate with the healthcare team, address social needs and create self-management plan.<br>**IG 2:** usual home care + 30-day transitional NP+ health coach (HC) for 60 days after NP visits finish. HC added 3 in-home visits and 3 telephone visits focusing on stroke prevention, supporting self-management, and facilitating reintegration into the community<br>**CG:** usual home care (nursing or rehab, patient education, monitoring, and social work)	**Primary Outcome Goal:** improved adherence to the care plan<br><br>**Measurement Method:** SBP
Mandigout et al. (Ticaa'dom)	**IG:** provided individualized diagnosis, selection of physical activities, goal setting, and a wrist activity monitor, followed by 6 months of (1) therapist and patient daily perceived physical activity measure, (2) weekly phone call from therapist to the patient about physical activity, (3) 3-weekly home visit for follow up and goal setting, followed by 6 months of usual care<br><br>**CG:** 6 months of usual care	**Primary Outcome Goal:** Improved exercise<br><br>**Measurement Method:** 6MWT<br><br>**Secondary Outcomes:** Motricity Index, ambulatory ability (mFAC), fat and muscle mass %, maximal strength knee extension
Dregan et al. (Point of Care EHR Prompt)	**IG:** Point of care secondary stroke prevention guideline prompt installed on EHR software which prompted physicians to follow guidelines. Also included EBM resources for physicians and printouts for patients.<br><br>**CG:** no prompt on EHR	**Primary Outcome Goal:** Improved adherence to care plan *at the physician level*<br><br>**Measurement Method:** BP, statin prescription (if cholesterol >4mmol/L), prescription for anticoagulant if eligible, prescription for antiplatelet drugs for AIS patients

Holmgren et al. (High-Intensity Exercise Program)	**IG:** 5-week program with 7 sessions/week divided over 3 days, 1 of which was a group discussion about falls risk, the remaining were supervised exercise sessions. Participants were also given a home exercise program for the other 4 days of the week.<br><br>**CG:** once weekly group discussions on hidden dysfunction and coping post-stroke for 5 weeks, specific topics of discussion chosen by participants, no focus on falls risk	**Primary Outcome Goal:** Improved education and exercise<br><br>**Measurement Method:** QoL Scale (SF-36) and Geriatric Depression Scale
Hornnes et al. (PREVENT)	**IG:** Home visits from a nurse at 1, 4, 7, and 10 months after randomization to have B measured, discuss medication compliance, and receive tailored lifestyle counseling. If BP was elevated patient was encouraged to attend GP for medication adjustment.<br><br>**CG:** standard of care	**Primary Outcome Goal:** Improved adherence to the care plan<br><br>**Measurement Method:** BP, stage of HTN, antihypertensive drug use, compliance with antihypertensive therapy, and visits to GP
Bretz et al. (STARS-Plus)	**IG:** supportive telephone calls regarding well-being and education needs, scheduled delivery of education materials (self-advocacy, caregiver well-being, and community re-integration), improved contact with healthcare providers<br>**CG:** none	**Primary Outcome Goal:** Improved adherence to healthcare plan and education<br><br>**Measurement Method:** Pre-post improvement in health status self-report (SF-12)<br><br>**Secondary Outcomes:** Medication adherence and resource utilization
Fukuoka et al. (DMP)	**IG:** Nurses worked with PCP to achieve individualized clinical targets and supplied patients with education booklets supporting RF control, plus a self-management journal (daily BP, body weight, and lifestyle goals). Abnormalities in self-monitored data were reported to PCP.<br><br>**CG:** standard of care + access to a patient information leaflet and self-management journal at discharge	**Primary Outcome Goal:** Improved adherence to care plan and education<br><br>**Measurement Method:** Framingham risk score<br><br>**Secondary Outcomes:** Stroke recurrence and vascular events
Maasland et al. (ICMP)	**IG:** Individualized multimedia computer program (1 session lasting 20-30 min) to provide info on general information and modifiable RFs. Individualization occurred at the level of health education and level of deviation from individual target parameters.<br>**CG:** standard of care	**Primary Outcome Goal:** Improved education<br><br>**Measurement Method:** Investigator created and validated knowledge questionnaire score at 1 week<br><br>**Secondary Outcome:** Knowledge questionnaire, mRS, changes in cholesterol, weight smoking, alcohol, and PA at 12 weeks
Eames et al. (Education & Support Package)	**IG:** Written information booklet, pre-discharge face-to-face education, and telephone follow up for 3 months after discharge<br>**CG:** standard of care	**Primary Outcome Goal:** Improved education<br><br>**Measurement Method:** Knowledge of Stroke questionnaire score<br><br>**Secondary Outcomes:**<br>Self-efficacy (investigator developed scale, not validated), anxiety and depression (HADS score), quality of life (SAQoL-39g score), satisfaction with information 10-point Likert scale, feeling informed 10-point Likert scale and Caregiver Strain Index score
Machline-Carrion et al. (BRIDGE Stroke)	**IG:**<br>• Case management (to ensure delivery of materials, implementation of materials, and support when needed	**Primary Outcome Goal:** improved adherence to healthcare plan and education *at the physician level*

	• Treatment algorithm reminders (patient wristbands, therapeutic plan attached to patient medical record)<br>• Education material (posters, tPA kit, bedside dysphagia screening test, NIH stroke scale and medication brochure for physicians and educational brochure for patients)<br>• Audit (feedback report sent to hospital)<br>• Interactive training workshops (simulation-based training and tailoring of intervention to needs of each site)<br><br>**CG:** standard of care	**Measurement Method:** Composite adherence score (sum of usage of evidence-based therapies in first 48 hours and at discharge – antithrombotic therapy, DVT prophylaxis, tPA door to needle time, dysphagia screening, rehab assessment, statins, anticoagulation, and smoking cessation)<br><br>**Secondary Outcomes:** All or none measures of individual elements of the composite primary outcome and hospital events
Boter et al. (Outreach Nursing)	**IG:** 3 nurse-initiated telephone contacts and 1 visit to home 10-14 weeks after discharge to run through the standardized checklist (RF, stroke consequences, and unmet needs). Patients were advised to attend GP if problems arose and were supplied with brochures where appropriate.<br><br>**CG:** standard of care	**Primary Outcome Goal:** improved adherence to the care plan<br><br>**Measurement Method:** Satisfaction with Stroke Care (SASC-19) questionnaire and QoL (SF-36)<br><br>**Secondary Outcomes:** anxiety and depression (HADS score), ability to perform ADL (Barthel index), independence (mRS), use of health care services since discharge, use of secondary prevention drugs since discharge) and caregiver data (caregiver strain index and sense of competence questionnaire)
Barker-Collo et al. (Motivational Interviewing)	**IG:** 4x30 min telephone or home motivational interview sessions at 28 days, 3, 6, and 9 months<br><br>**CG:** standard of care	**Primary Outcome Goal:** Improved adherence to the care plan<br><br>**Measurement method:** SBP and LDL-C<br><br>**Secondary Outcomes:** HDL-C, total cholesterol, new vascular events, QoL (SF-36), mood (HADS), and medication adherence

Table S2. Ongoing, pilot, and feasibility study summary

**Study Title, Location, and N**	**Study Design**<br>**Intervention Level**	**Delivered by**	**Target group**	**Primary Outcome**	**Strategy**<br>*(IG = intervention group*<br>*CG = Control Group)*
Telemedicine Guided Education[42]<br>USA<br>N predicted = 50	Single-arm feasibility<br>Level 1 intervention	Videoconference-delivered	Stroke patients	Recruitment, compliance, satisfaction	**IG:** 7-week telerehabilitation program on a loaned iPad<br>**CG:** none
StrokeCoach[43]<br>Canada<br>N=126	RCT<br>Level 1 intervention	Lifestyle coach-delivered	Stroke patients	Health Promoting Lifestyle Profile II measures at 6 months	**IG:** 7 telephone lifestyle and memory training coaching sessions<br>**CG:** standard of care

CEOPS[44]<br>France<br>N=410	RCT<br>Level 1 intervention	Nurse-delivered	Stroke or TIA patients	BP at 24 months	**IG:** 2 years of phone calls with vascular nurse<br>**CG:** standard of care
SPRITE[45]<br>Northern Ireland<br>N=28	Randomized feasibility<br>Level 1	Written material	Stroke or TIA patients	Recruitment	**IG:** Cardiac home-based rehabilitation manual ± pedometer<br>**CG:** standard of care
PREVENT[46]<br>Canada<br>N=250	RCT<br>Level 1 intervention	HCW-delivered	Stroke or TIA patients	Blood pressure, waist circumference, fasting lipids, fasting glucose, HbA1cat 12 months	**IG:** 12-week exercise and education program<br>**CG:** standard of care
A Structured Patient-Centered Educational Exchange[47,48]<br>Australia N=16	Single-arm feasibility<br>Level 1 intervention	HCW-delivered	Stroke patients	Feasibility	**IG:** Patient-centered face to face discussion at the bedside before discharge and a telephone follow up<br>**CG:** standard of care
SystemCHANGE and Attention Control[41]<br>USA<br>N=2	RCT<br>Level 1 intervention	Primary investigator-delivered	Non-medication-adherent stroke patients	Medication adherence<br>Feasibility	**IG 1 (SystemCHANGE):** Assistance changing daily routines<br>**IG 2 (Attention Control):** Educational materials on stroke<br>**CG:** none

Table S3. PRISMA Checklist

Section and Topic	Item #	Checklist item	Location where item is reported
**TITLE**			
Title	1	Identify the report as a systematic review.	Title
**ABSTRACT**			
Abstract	2	See the PRISMA 2020 for Abstracts checklist.	n/a
**INTRODUCTION**			
Rationale	3	Describe the rationale for the review in the context of existing knowledge.	Introduction
Objectives	4	Provide an explicit statement of the objective(s) or question(s) the review addresses.	Introduction
**METHODS**			
Eligibility criteria	5	Specify the inclusion and exclusion criteria for the review and how studies were grouped for the syntheses.	Methods
Information sources	6	Specify all databases, registers, websites, organisations, reference lists and other sources searched or consulted to identify studies. Specify the date when each source was last searched or consulted.	Methods
Search strategy	7	Present the full search strategies for all databases, registers and websites, including any filters and limits used.	Methods

Selection process	8	Specify the methods used to decide whether a study met the inclusion criteria of the review, including how many reviewers screened each record and each report retrieved, whether they worked independently, and if applicable, details of automation tools used in the process.	Methods
Data collection process	9	Specify the methods used to collect data from reports, including how many reviewers collected data from each report, whether they worked independently, any processes for obtaining or confirming data from study investigators, and if applicable, details of automation tools used in the process.	Methods
Data items	10a	List and define all outcomes for which data were sought. Specify whether all results that were compatible with each outcome domain in each study were sought (e.g. for all measures, time points, analyses), and if not, the methods used to decide which results to collect.	Methods
	10b	List and define all other variables for which data were sought (e.g. participant and intervention characteristics, funding sources). Describe any assumptions made about any missing or unclear information.	Methods
Study risk of bias assessment	11	Specify the methods used to assess risk of bias in the included studies, including details of the tool(s) used, how many reviewers assessed each study and whether they worked independently, and if applicable, details of automation tools used in the process.	Methods
Effect measures	12	Specify for each outcome the effect measure(s) (e.g. risk ratio, mean difference) used in the synthesis or presentation of results.	N/a
Synthesis methods	13a	Describe the processes used to decide which studies were eligible for each synthesis (e.g. tabulating the study intervention characteristics and comparing against the planned groups for each synthesis (item #5)).	N/a
	13b	Describe any methods required to prepare the data for presentation or synthesis, such as handling of missing summary statistics, or data conversions.	N/a
	13c	Describe any methods used to tabulate or visually display results of individual studies and syntheses.	N/a
	13d	Describe any methods used to synthesize results and provide a rationale for the choice(s). If meta-analysis was performed, describe the model(s), method(s) to identify the presence and extent of statistical heterogeneity, and software package(s) used.	N/a
	13e	Describe any methods used to explore possible causes of heterogeneity among study results (e.g. subgroup analysis, meta-regression).	N/a
	13f	Describe any sensitivity analyses conducted to assess robustness of the synthesized results.	N/a
Reporting bias assessment	14	Describe any methods used to assess risk of bias due to missing results in a synthesis (arising from reporting biases).	N/a
Certainty assessment	15	Describe any methods used to assess certainty (or confidence) in the body of evidence for an outcome.	N/a
**RESULTS**			
Study selection	16a	Describe the results of the search and selection process, from the number of records identified in the search to the number of studies included in the review, ideally using a flow diagram.	Results
	16b	Cite studies that might appear to meet the inclusion criteria, but which were excluded, and explain why they were excluded.	Results
Study characteristics	17	Cite each included study and present its characteristics.	Table S2

			Table 2<br>Table 3
Risk of bias in studies	18	Present assessments of risk of bias for each included study.	N/a
Results of individual studies	19	For all outcomes, present, for each study: (a) summary statistics for each group (where appropriate) and (b) an effect estimate and its precision (e.g. confidence/credible interval), ideally using structured tables or plots.	N/a
Results of syntheses	20a	For each synthesis, briefly summarise the characteristics and risk of bias among contributing studies.	N/a
	20b	Present results of all statistical syntheses conducted. If meta-analysis was done, present for each the summary estimate and its precision (e.g. confidence/credible interval) and measures of statistical heterogeneity. If comparing groups, describe the direction of the effect.	N/a
	20c	Present results of all investigations of possible causes of heterogeneity among study results.	N/a
	20d	Present results of all sensitivity analyses conducted to assess the robustness of the synthesized results.	N/a
Reporting biases	21	Present assessments of risk of bias due to missing results (arising from reporting biases) for each synthesis assessed.	N/a
Certainty of evidence	22	Present assessments of certainty (or confidence) in the body of evidence for each outcome assessed.	N/a
**DISCUSSION**			
Discussion	23a	Provide a general interpretation of the results in the context of other evidence.	Discussion
	23b	Discuss any limitations of the evidence included in the review.	Discussion
	23c	Discuss any limitations of the review processes used.	Discussion
	23d	Discuss implications of the results for practice, policy, and future research.	Discussion
**OTHER INFORMATION**			
Registration and protocol	24a	Provide registration information for the review, including register name and registration number, or state that the review was not registered.	N/a
	24b	Indicate where the review protocol can be accessed, or state that a protocol was not prepared.	N/a
	24c	Describe and explain any amendments to information provided at registration or in the protocol.	N/a
Support	25	Describe sources of financial or non-financial support for the review, and the role of the funders or sponsors in the review.	N/a
Competing interests	26	Declare any competing interests of review authors.	N/a
Availability of data, code and other materials	27	Report which of the following are publicly available and where they can be found: template data collection forms; data extracted from included studies; data used for all analyses; analytic code; any other materials used in the review.	N/a

Figure S1 PRISMA Flow Chart

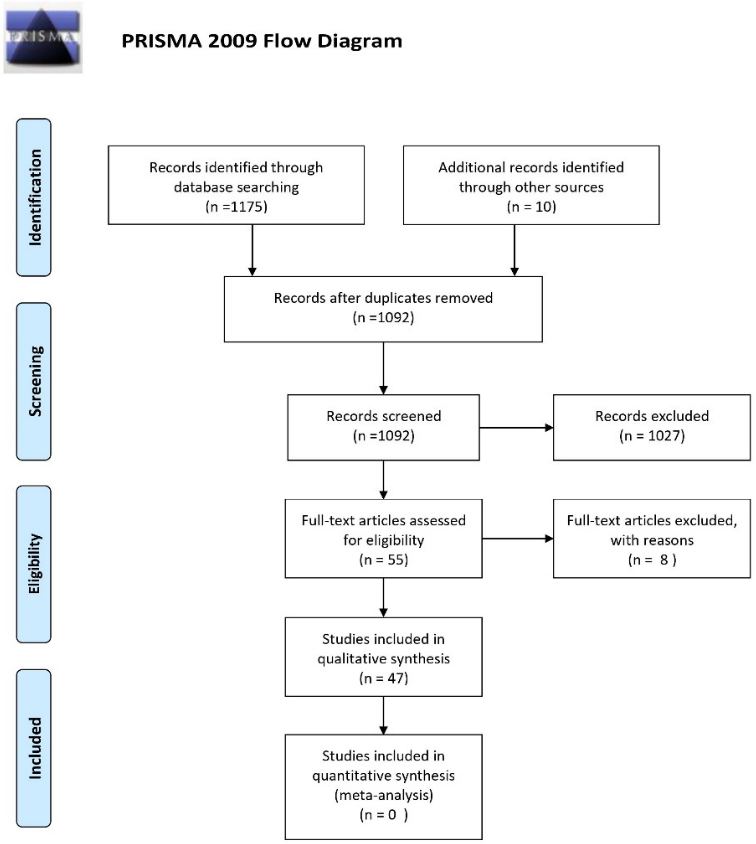

From: Moher D, Liberati A, Tetzlaff J, Altman DG, The PRISMA Group (2009). *P*referred *R*eporting *I*tems for *S*ystematic Reviews and *M*eta-*A*nalyses: The PRISMA Statement. PLoS Med 6(7): e1000097. doi:10.1371/journal.pmed1000097

For more information, visit www.prisma-statement.org.
